# Midlife physical activity is associated with lower incidence of vascular dementia but not Alzheimer’s disease

**DOI:** 10.1186/s13195-019-0538-4

**Published:** 2019-10-20

**Authors:** Oskar Hansson, Martina Svensson, Anna-Märta Gustavsson, Emelie Andersson, Yiyi Yang, Katarina Nägga, Ulf Hållmarker, Stefan James, Tomas Deierborg

**Affiliations:** 1 Clinical Memory Research Unit, Department of Clinical Sciences Malmö, Lund University, Malmö, Sweden; 2 Memory Clinic, Skåne University Hospital, Malmö, Sweden; 3 Experimental Neuroinflammation Laboratory, Department of Experimental Medical Science, Lund University, 221 84 Lund, Sweden; 4 Department of Acute Internal Medicine and Geriatrics, Linköping University, Linköping, Sweden; 5 Department of Medical Sciences, Cardiology, Uppsala University, Uppsala, Sweden

**Keywords:** Physical activity, Alzheimer’s disease, Vascular dementia, Exercise, Amyloid-β

## Abstract

**Background:**

Physical activity might reduce the risk of developing dementia. However, it is still unclear whether the protective effect differs depending on the subtype of dementia. We aimed to investigate if midlife physical activity affects the development of vascular dementia (VaD) and Alzheimer’s disease (AD) differently in two large study populations with different designs.

**Methods:**

Using a prospective observational design, we studied whether long-distance skiers of the Swedish Vasaloppet (*n* = 197,685) exhibited reduced incidence of VaD or AD compared to matched individuals from the general population (*n* = 197,684) during 21 years of follow-up (median 10, interquartile range (IQR) 5–15 years). Next, we studied the association between self-reported physical activity, stated twice 5 years apart, and incident VaD and AD in 20,639 participants in the Swedish population-based Malmo Diet and Cancer Study during 18 years of follow-up (median 15, IQR 14–17 years). Finally, we used a mouse model of AD and studied brain levels of amyloid-β, synaptic proteins, and cognitive function following 6 months of voluntary wheel running.

**Results:**

Vasaloppet skiers (median age 36.0 years [IQR 29.0–46.0], 38% women) had lower incidence of all-cause dementia (adjusted hazard ratio (HR) 0.63, 95% CI 0.52–0.75) and VaD (adjusted HR 0.49, 95% CI 0.33–0.73), but not AD, compared to non-skiers. Further, faster skiers exhibited a reduced incidence of VaD (adjusted HR 0.38, 95% CI 0.16–0.95), but not AD or all-cause dementia compared to slower skiers. In the Malmo Diet and Cancer Study (median age 57.5 years [IQR 51.0–63.8], 60% women), higher physical activity was associated with reduced incidence of VaD (adjusted HR 0.65, 95% CI 0.49-0.87), but not AD nor all-cause dementia. These findings were also independent of *APOE*-ε4 genotype. In AD mice, voluntary running did not improve memory, amyloid-β, or synaptic proteins.

**Conclusions:**

Our results indicate that physical activity in midlife is associated with lower incidence of VaD. Using three different study designs, we found no significant association between physical activity and subsequent development of AD.

## Background

Alzheimer’s diseases (AD) followed by vascular dementia (VaD) are the most common types of dementia. Risk factor control is an important strategy to postpone dementia onset, and physical inactivity is regarded as one of the main modifiable risk factors that can be targeted [1, 2]. However, recent intervention trials involving physical activity report mixed results, thereby highlighting the lack of consistency within the field [3–5]. A systematic review showed that physical activity interventions improved cognition in demented persons [6], but revealed that most trials do not distinguish between pure AD and pure VaD patients. Published trials are often multi-domain interventions, making it difficult to draw any conclusions regarding the effect of only physical activity. Among trials with physical activity as the only intervention, improved cognition was reported in patients with mild AD after 16 weeks of exercise [7], whereas no cognitive effects were seen in demented patients after 12 months [8]. An ongoing trial with a 2-year exercise intervention will provide further information if physical exercise can be beneficial in preventing dementia [9]. As summarized in reviews and meta-analyses, findings from previous prospective cohort studies *differ* but pooled results indicate protective effects [10–14]. Nevertheless, there are important concerns within the prevailing literature, such as possible publication bias and follow-up effects [11].

Beneficial effects are mainly found in late-life assessments with short-term follow-up [10, 13, 15, 16] and tend to become non-significant after longer follow-up [11, 15, 16]. These discrepancies may be attributable to reverse causation where cognitive dysfunction may lead to reduced physical activity. A recent population-based study on physical activity and dementia (*n* = 10,308) provide repeated physical activity assessments and reports that physical activity begins to decline up to 9 years before diagnosis of dementia [17], thus emphasizing the possible impact of reverse causation in studies with shorter follow-up. In this study, no association between midlife physical activity and dementia was found during 27 years of follow-up [17].

Further, the different diseases causing cognitive impairment are associated with very different underlying disease mechanisms, such as gradual accumulation of amyloid-β (Aβ) and tau in AD and arteriosclerosis and ischemia in VaD. Therefore, it is unlikely that the same preventive strategies are equally effective against different pathological mechanisms causing dementia. Literature reports variable effects of physical activity on incident VaD [12, 18] and AD [15, 18–21]. Furthermore, it is unclear whether individuals carrying the genetic risk factor *APOE-*ε4 [22] might benefit specifically from physical exercise [16, 23, 24]. Working in transgenic animal models makes it easier to study the mechanistic effects of physical activity on different molecular hallmarks of AD, such as Aβ and synaptic proteins, as well as cognitive symptoms. Indeed, several studies have been conducted to investigate the effect of physical activity on AD pathology [25]. For example, exercise resulted in improved cognition [25] as well as reduction of both Aβ soluble and insoluble Aβ species in a dose-dependent manner [26]. However, the effects are inconsistent between studies [25], since other studies show lack of effects [25, 27]. The majority of studies also investigate the effect of physical activity in a relatively short period of time [25]. Thus, additional experimental studies are needed to investigate the long-term effects of physical activity, starting in the pre-manifest stage, on AD hallmarks.

As mentioned, the setup and quality of published studies in the field are limited [11, 13]. Long follow-up periods are needed to reduce the effect of reverse causation, and large study populations are necessary to study differences between dementia subtypes. To address these limitations, we investigated if physical activity in midlife affects the development of VaD and AD in two separate large study populations with different study designs and long follow-up. Further, to study the long-term effect on AD pathology such as Aβ and synaptic proteins, we exposed transgenic AD mice to voluntary wheel running.

## Materials and methods

### Dementia diagnoses

Dementia diagnoses were made by physicians in clinical routine and retrieved from the Swedish National Patient Register (NPR). It started in 1964, and since 1987, it provides information on all primary and secondary diagnoses, covering 99% of all hospital-based diagnoses. Primary care diagnoses are not included. Dementia was defined as any dementia diagnosis according to the International Classification of Diseases, tenth revision or ninth revision. Diagnoses included are AD (F00, G30, 331A/3310, 29010), VaD (F01, 290E/2904), or other forms included among all-cause dementia (2900, 2901, F023, 2941/294B, 3320/332A, F028, G318A, 331/331X, 33182/331H, F020, G310, 3311/331B, F03, F070, 290, or 2942/294C). Based on this classification, AD cases include atypical and mixed cases (F002), thus also covering AD with a vascular component. In the Vasaloppet cohort, the differentiation between AD and VaD was done by the diagnosing physician in line with the available clinical diagnostic criteria and no further information on the diagnostic routine was available. In the Malmo Diet and Cancer study (MDCS), we reviewed and verified all register diagnoses in medical records as part of the research protocol. Among MDCS dementia cases (*n* = 1375), electronic charts provided history regarding cognitive symptoms in 92%, cognitive test results in 92%, and neuroimaging (mainly CT) in connection to diagnosis in 99.6%, which were all reviewed by us in depth to determine the type of dementia diagnosis (see below). Further, 82% were assessed at a tertiary unit specializing in memory disorders, where CSF analyses of AD biomarkers were often part of the diagnostic work-up.

### Vasaloppet cohort

#### Physical activity

The Vasaloppet study population comprises non-demented participators of the world’s largest long-distance (30 to 90 km) cross-country ski race (Vasaloppet) between 1989 and 2010 (*n* = 197,685), together with frequency-matched, non-demented individuals from the general population (*n* = 197,684). Frequency matching was done from the population register according to age group (5-year intervals), sex, region of residency, and year of participation in ski race as previously described [28]. In the first matching process, a control individual from the general population was assigned for every ski race, so that skiers participating in Vasaloppet several times got several controls. We performed a re-matching procedure to get equally many skiers as non-skiers. Since we only used the index race for each skier, the non-skiers would have been older as a group if we had included one control for every time a skier participated in the race. The total study cohort (*n* = 395,369) was prospectively followed in the Swedish NPR throughout 2010. Skiers are considered to be physically active since it is necessary to undergo regular physical training in order to complete such a demanding long-distance race. For example, the majority of skiers exercise for at least 4 h a week [29]. On average, Vasaloppet skiers have higher leisure time physical activity than the general Swedish population [30]. Regarding fitness, the oxygen consumption (V_O2MAX_) has been shown to be 45–80 ml/kg/min in skiers, compared to around 35 ml/kg/min in the general population [31].

#### Covariates

Information on date of birth, sex, and education level was derived from Swedish registries [28]. We categorized education as primary/elementary school (≤ 8 years), secondary school/high school (9–12 years), or higher education/university (≥ 13 years). No further data were available in this cohort.

#### Attrition

**Fig. 1:**
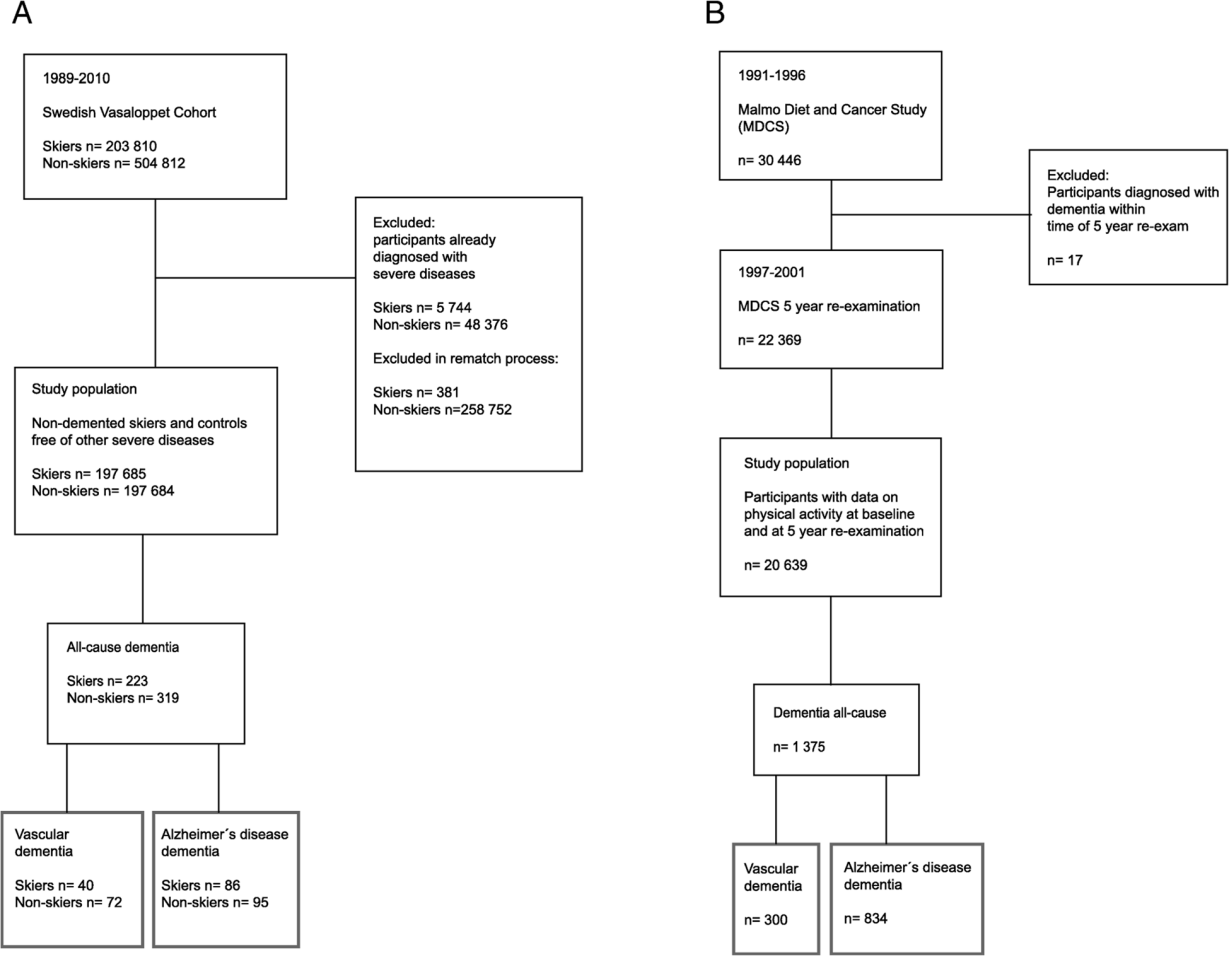
Vasaloppet and MDCS study populations. Flow diagram describing the Vasaloppet Study population (**a**) and MDCS population (**b**)

In addition to having higher physical activity, the average Vasaloppet skier also smokes less and has a healthier diet and lower mortality than the general Swedish population [30]. To avoid bias due to inability to participate in the race because of poor health, individuals with severe disease were excluded as previously described [31]. We additionally excluded participants with Parkinson’s disease (G20, 332A, 3420), meningitis/encephalitis (G00, G01, G03, G04, G05, 3200, 320A, 320B, 320C, 320D, 320 W 320X, 321A, 321B, 321C, 321D, 321E, 321X, 322A, 322B, 322C, 320X, 323, 3230), epilepsy (G40, 345, 3450), depressive episode (F32, F33, F34, F38, F399, 296B, 296X, 29620, 29800), manic episode (F30, F29, 296A, 29610), bipolar disorder (F310, F311, F312, F313, F314, F315, F316, F317, F318, F319, 296C, 296D, 296E, 29600, 29610, 29620, 29630, 29688, 29699), anxiety disorders (F40, F41, F42, 300A, 300B, 300C, 300D, 300D, 3000, 3001, 3002, 3003), and mental disorders due to the use of alcohol (F10, 291, 2910, 2919). A flow diagram describing numbers excluded can be seen in Fig. 1a.

### Malmo Diet and Cancer study (MDCS) cohort

The MDCS population is part of a large prospective population-based study, where baseline investigations were performed between 1991 and 1996. At baseline, participants responded to questionnaires and underwent a basic clinical examination. Research nurses draw blood samples and measured height, weight, and blood pressure [32]. Five years later, between 1997 and 2001, participants were invited to respond to the questionnaire again as part of a reexamination. The present study cohort (*n* = 20,639) consists of all participants who were non-demented at the reinvestigation and provided data on physical activity at both baseline and reinvestigation (Fig. 1b).

#### Physical activity assessment

Information on physical activity during leisure time was stated in both questionnaires as the form of physical activity (e.g., walking, gardening, and running) and minutes per week the activity was performed at every season (spring, summer, autumn, winter). The activity was multiplied with an activity-specific factor, where heavier activities were graded with a higher factor [33]. This generated a total physical activity score calculated as the sum of minutes per week for all four seasons multiplied with the activity-specific factor, for every activity stated. We calculated the combined physical activity score as the sum of the scores from the two time points.

#### Review of dementia diagnoses

The MDCS cohort was followed in the Swedish NPR throughout 2014, when all registered dementia diagnoses were extracted. A diagnostic evaluation was performed by medical doctors at the Memory Clinic at Skåne University Hospital. All register diagnoses were reviewed in medical records and evaluated based on symptom presentation, test results, and brain imaging in accordance with DSM-5 (The Diagnostic and Statistical Manual of Mental Disorder, Fifth Edition) [34]. One thousand four hundred forty-six dementia diagnoses were first identified in the register. Based on the diagnostic review process, 54 out of 1446 individuals (3.7%) did not meet criteria for dementia (e.g., reversible disorientation, major depression, or mild cognitive impairment) and were instead regarded as non-demented participants. Further, 17 out of 1446 individuals (1.2%) received their dementia diagnosis within the time of the reinvestigation and were excluded (Fig. 1b). Among the 20,639 participants in the final study population, 1375 individuals (6.7%) fulfilled the criteria for dementia. The diagnosis was refined in 322 of 1446 cases (22%), mainly from unspecified dementia to AD with concomitant vascular disease. One hundred three participants (7%) remained classified as unspecified dementia since available medical records did not provide enough information to diagnose with further accuracy. In 172 individuals, no e-chart was available (mainly due to emigration or death before conversion to the current e-chart system) and then the last diagnosis in the register was used.

#### Covariates

Covariates were selected based on previous literature and availability [35]. Information on education, smoking, alcohol consumption, medication use, and work-related physical activity was self-reported and derived from the baseline questionnaire. We categorized education as primary/elementary school (≤ 8 years), secondary school/high school (9–12 years), or higher education/university (≥ 13 years). Smoking was categorized as ever smoker (current or former) or never smoker. Alcohol consumption was entered numerically as grams of alcohol per day, computed from the units of beer, wine, and liquor participants stated to have consumed during the last month. Drugs were classified according to the international Anatomical Therapeutic Chemical Classification (ATC). Blood pressure-lowering medication was defined as any drug with blood pressure-lowering effect regardless of indication and consisted of diuretics (ATC group C03), beta-blocking agents (ATC group C07), calcium channel blockers (ATC group C08), or agents acting on the renin-angiotensin system (ATC group C09). Lipid-lowering medication was defined as any drug with serum lipid-reducing effect (ATC group C10). Work activity was stated as “what degree of physical activity is usually demanded in your work” with options (1) very light, (2) light or medium heavy, (3) heavy, or (4) very heavy. We categorized heavy or very heavy as physically heavy work. Baseline information on the prevalence of diabetes mellitus (type 1 or 2) was derived from the Swedish National Diabetes Register and the NPR. Cardiovascular disease was defined as ischemic or hemorrhagic stroke or ischemic heart disease and originates from the NPR and the Stroke register of Malmo.

#### Attrition

In the MDCS database, there are data on 30,446 individuals. When comparing baseline data for participants included in the present study (*n* = 20,639) and the remaining original cohort (*n* = 9807), included participants were younger (mean age [SD] 57.8 [7.5] years vs 58.5 [7.8] years, *p* < 0.001) and had a higher physical activity score at baseline (mean score 8292 [6746] vs 7532 [6344], *p* < 0.0001). Further, included participants were higher educated and generally healthier (e.g., had lower blood pressure, less cardiovascular disease, and less diabetes) than non-included individuals (*p* < 0.0001). Further, the incidence rate per 1000 person-years (based on time from baseline till event or end of study) differs between included participants (3.5 for any dementia, 0.8 for VaD, and 2.1 for AD) and non-participants (4.7 for any dementia, 1.5 for VaD, and 2.4 for AD). There were no differences in sex or *APOE*-ε4 carrier status. Further information on recruitment bias has been described in previous publications [36].

### 5xFAD mouse model

The 5xFAD strain is a mouse model co-expressing five mutations associated with familial form of AD, resulting in increased production of Aβ42. These mice have a fast development of AD pathology, showing accumulation of Aβ plaques as early as 2–3 months of age, cognitive dysfunctions already at 5 months of age, and neuronal and synaptic losses at 9 months of age [37–39]. Taken together, this makes the 5xFAD a suitable mouse model to study the effects of exercise on the development of Aβ plaque load, as well as cognitive dysfunctions seen in AD patients.

We used female 5xFAD mice (*n* = 30), aged 9–12 weeks, from Jackson Laboratories, weighing 14–20 g when starting the experiment. Mice were housed two animals/cage in standard laboratory cages with sawdust bedding and free access to water and food. They acclimatized for at least 5 days before starting the experiment. The holding room had a 12:12 h light-dark cycle. There were no differences in body weight, age, and general motor function between the groups when the experiment was initiated.

#### Voluntary running wheel exercise

Mice were randomly assigned to sedentary (*n* = 14) or exercising (*n* = 16) group. At 9–12 weeks of age, mice in the running group were provided with low-profile wireless running wheels for mouse (ENV-047; med-associates.com) in their home cage, allowing the mice to run as much as and whenever they wanted, during 24 weeks, until the end of the study.

#### Cognitive tests

Y-maze spontaneous alternation test was performed to examine any defects in working-memory after 18 weeks of running as previously described [40]. For this purpose, a Y-maze arena (21 × 4 cm/arm) was used. Mice with less than five arm entries were excluded from the analysis. Y-maze spatial memory test was performed to examine any defects in hippocampus-dependent spatial memory after 21 weeks of running as described previously [41]. To examine hippocampus-independent object memory, the mice were subjected to a novel object recognition test after 19–20 weeks of running. This test was conducted in an open field arena (30 cm × 30 cm) as described previously [42]. Both training and trial session duration was 5 min. Mice that did not explore both objects at least one time during the trial session were excluded.

#### Collection of samples

After 24 weeks of running, samples were collected. The mice were anesthetized with isofluorane and perfused with saline solution before the brains were dissected out. The right hemisphere was fixed in 4% paraformaldehyde in phosphate buffer for 24 h before they were stored in 30% sucrose solution at 4 °C until analysis. From the left hemisphere, the hippocampus and cortex were dissected, snap frozen on dry ice, and stored at − 80 °C until analysis.

#### Western blot

The hippocampus was homogenized as previously described [43] with some modifications. Briefly, we used 120 μl of TBS buffer (20 mM Tris-HCl, 137 mM NaCl, pH 7.6) containing protease and phosphatase inhibitors and 1% Triton-X100 in a dounce homogenizer. After 30-min incubation on ice, it was centrifuged at 14000*g* at 4 °C for 30 min. The supernatant was collected. Protein concentrations were determined (Pierce microplate BCA Protein Assay kit, thermofisher.com). Western blot was used as previously described [44]. The levels of the synaptic proteins PSD-95 (1:3000, MAB1596, Millipore) and synaptophysin (1:1000, Ab14692, Abcam,) were measured and normalized to beta-actin.

#### Immunohistochemistry

Immunohistochemistry was performed as previously described [44] with some modifications. Briefly, 30-μm sagittal sections were stained with 6E10 (1:500; BioLegend, San Diego, USA) and secondary antibody labeled with Alexa Fluor® 594 (1:500; Invitrogen, Carlsbad, CA, USA). Three sections per brain (lateral 0.84–1.2 mm) were analyzed using an epifluorescence microscope (Nikon Eclipse 80i microscope, Europe). The 6E10-positive Aβ were analyzed in dentate gyrus/CA4 in the hippocampus and cortical layer 4 and 5 in the neocortex area above/dorsally of the lateral ventricle. The immunofluorescence intensity was measured in 0.25 mm^2^ within regions of interest using ImageJ.

#### ELISA

The concentration of Aβ species (Aβ40 and Aβ42) in the homogenized hippocampus was measured as previously described [44], with the MSD MULTI-SPOT Human (4G8) Aβ Assay (K15199G-1, Mesoscale) using QuickPlex SQ120 (Mesoscale Discovery, Rockville, USA) Plate Reader according to the manufacturer’s instructions. The recorded data was analyzed using MSD Discovery Workbench software. Aβ concentrations were normalized to total protein concentrations measured in the BCA or Bradford assay.

### Statistical analyses

We used R statistical software and SPSS statistical software (v.22, Windows). Two-tailed *p* values < 0.05 were considered statistically significant. Demographic data are presented as median and interquartile range (IQR) or numbers (*n*) and percent (%). Numeric and categorical group differences were estimated with Mann-Whitney *U* test and Pearson’s *χ*^2^ test, respectively. Based on tertiles, participants in the MDCS were divided into three groups according to their reported physical activity in leisure time, referred to as high, intermediate, and low. Cox regression models were used to compare risk of dementia for skiers vs non-skiers in the Vasaloppet cohort and per SD increase in physical activity score (continuous variable converted to *z*-score) and per physical activity group (categorical variable) in the MDCS cohort. Time of event was defined as the date of first registered dementia diagnosis in the NPR. Censoring appeared when subjects died or at the time of register outtake/end of follow-up. In the Vasaloppet cohort, the time variable was calculated as years between participation in the ski race and event/censoring. In the MDCS cohort, the time variable was calculated as years between the reinvestigation and event/censoring since individuals who were diagnosed with dementia before the reinvestigation were excluded (i.e., no events occurred between baseline and reinvestigation based on the study design). Information on the date of death for deceased study individuals was available through Statistics Sweden and the Causes of Death Register, held at the National Board of Health and Welfare. In the MDCS, we also performed analyses treating death as a competing risk event, using the cmprsk (competing risk) package in R.

Risk of all-cause dementia, VaD and AD are presented as hazard ratios (HR) with 95% confidence intervals (CI). In the Vasaloppet cohort, we present both a crude model and an age-, sex-, and education-adjusted model (model 1). Education is categorized as noted in Table 1. In the MDCS cohort, adjustments were performed in a stepwise manner, where model 1 is adjusted for age, sex, and education and model 2 is further adjusted for smoking, systolic blood pressure, body mass index, alcohol consumption, diabetes, cardiovascular disease, blood pressure-lowering medication, lipid-lowering medication, and physically heavy work.

**Table 1:** Characteristics of the Vasaloppet study population

	All	Skiers	Non-skiers
	*n* = 395,369	*n* = 197,685	*n* = 197,684
Characteristics 1989–2010	Median (IQR) or *n* (%)	Median (IQR) or *n* (%)	Median (IQR) or *n* (%)
Age at baseline, years	36.0 (29.0–46.0)	36.0 (29.0–46.0)	36.0 (29.0–46.0)
Women	149,796 (38)	74,897 (38)	74,899 (38)
Education			
Primary/elementary school (≤ 8 years)	49,344 (13)	14,538 (7.4)	34,806 (18)***
Secondary school/high school (9–12 years)	176,571 (45)	76,635 (39)	99,936 (51)
Higher education/university (≥ 13 years)	166,133 (42)	106,147 (54)	59,986 (31)
Dementia diagnoses at follow-up	*N* events (incidence rate/1000 person-years)		
All-cause dementia	542 (0.14)	223 (0.11)	319 (0.16)
Vascular dementia	112 (0.03)	40 (0.02)	72 (0.04)***
Alzheimer’s disease dementia	181 (0.05)	86 (0.04)	95 (0.05)

Characteristics of the Vasaloppet study population presented for the whole cohort and by skiers and non-skiers separately

***p < 0.001. Group difference between skiers and non-skiers, estimated with Mann-Whitney *U* test (numeric variables) and Pearson’s *χ*^2^ test (categorical variables). Only significant differences are noted in the table

**Fig. 2:**
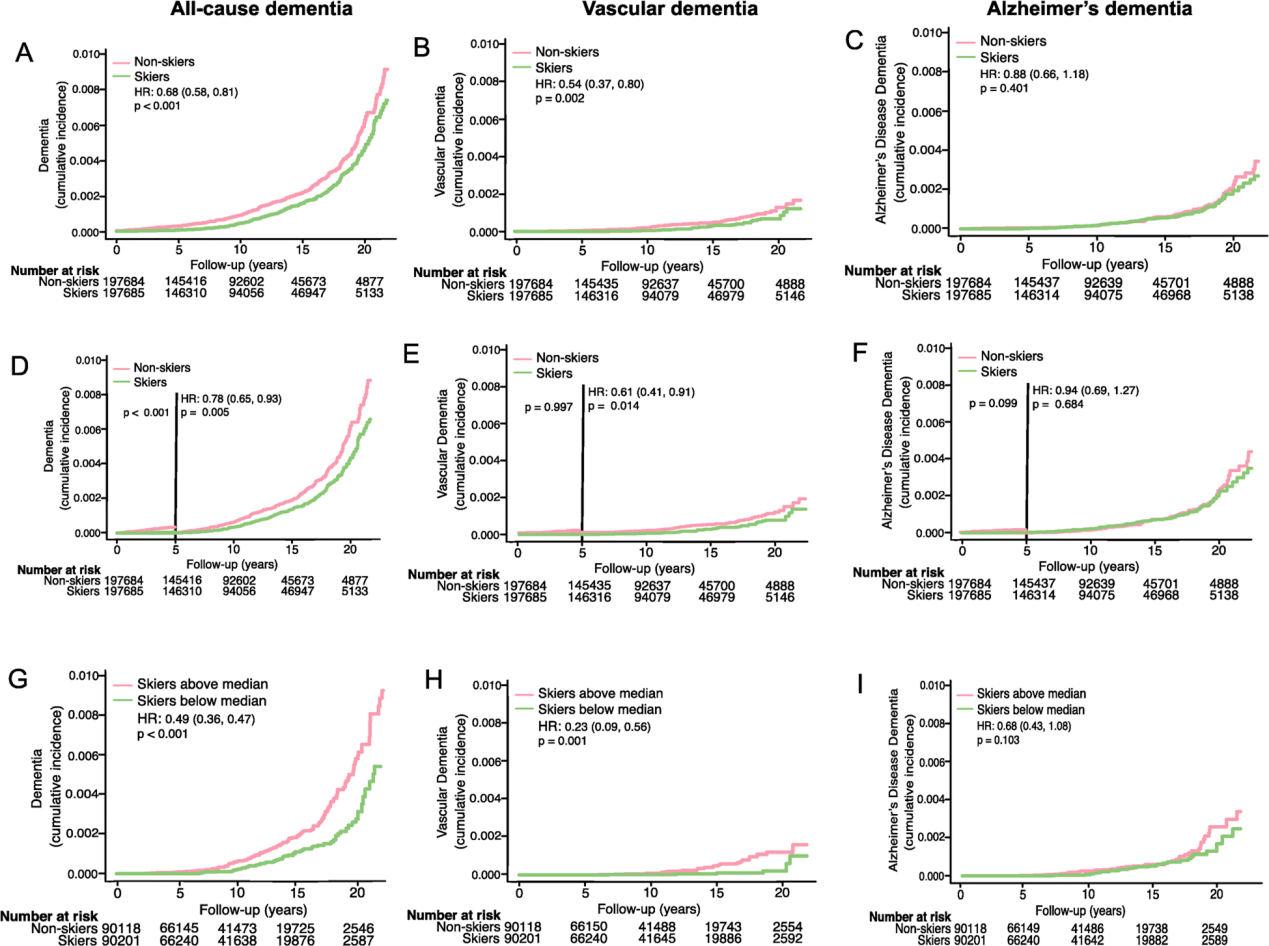
The effects of physical activity on the incidence of dementia, VaD, and AD in the Vasaloppet. The risk of developing all-cause dementia (**a**), VaD (**b**), or AD (**c**). The risk of developing all-cause dementia (**d**), VaD (**e**), or AD (**f**) more than 5 years after completing Vasaloppet. The risk of developing all-cause dementia (**g**), VaD (**h**), or AD (**i**) in skiers completing the Vasaloppet at a finishing time above or below median. HR represents hazard ratios from an unadjusted Cox regression

Overall, we performed complete case analyses, rendering fewer individuals in adjusted models. We modeled Schoenfeld residuals graphically to confirm the proportionality assumption. Figure data were constructed using Kaplan-Meier curves. The same time and event variables were used as in the Cox regressions, and the hazards are presented for skiers vs non-skiers. Numbers at risk were derived from survival tables specifying the number of individuals entering each 5-year interval, as presented in the graph (Fig. 2).

Since physical activity has been shown to be reduced up to 9 years before diagnosis [17] and beneficial effects of physical activity on dementia was shown to disappear after 4 years [15], we decided to set 5 years as a cut-off for sensitivity analyses. All individuals who developed dementia within 5 years of participation in the Vasaloppet ski race and within 5 years of the second physical activity assessment in the MDCS were excluded. In the MDCS, we performed further sensitivity analyses where we used pure AD and AD with cerebrovascular disease as separate event variables. We also added *APOE-*ε4 as a covariate in the subpopulation with available data and stratified this subpopulation on *APOE-*ε4 carrier status. Interaction statistics for *APOE-*ε4 was applied by simultaneously entering physical activity score and *APOE-*ε4 together with a variable consisting of their product in Cox regression models. In order to account for attrition bias, we also investigated if the physical activity at baseline (only one assessment) was associated with the different event variables (all-cause dementia, VaD, and AD).

## Results

### Vasaloppet skiers had a reduced risk of developing vascular dementia but not Alzheimer’s dementia

Demographic data for the Vasaloppet cohort is presented in Table 1. The total number of deaths was less than 2%. After a median follow-up of 10 years (IQR 5–15 years), 542 dementia diagnoses were identified in the NPR. Out of these, 112 (21%) were diagnosed with VaD and 181 individuals (33%) with AD. Participation in the Vasaloppet ski race was associated with a lower risk of developing all-cause dementia and VaD, but there was no significant difference between skiers and non-skiers for AD (Table 2, Fig. 2a–c). Skiers had higher education than non-skiers (Table 1), but adjustments for age, gender, and education did not alter the results (model 1, Table 2). When we excluded cases that developed dementia within 5 years of the ski race (baseline), results were not altered (Table 2, Fig. 2d–f). Furthermore, faster skiers (accomplishing Vasaloppet with a finishing time below median) had a lower incidence of VaD (adjusted hazard ratio (HR) 0.38, 95% CI 0.16–0.95), but not all-cause dementia (HR 0.80, 95% CI 0.59–1.09) or AD (HR 1.17, 95% CI 0.73–1.88), compared to slower skiers (Fig. 2g–i, unadjusted HR).

**Table 2:** Association between physical activity and incident dementia in the Vasaloppet cohort

	All-cause dementia	*p*	Vascular dementia	*p*	Alzheimer’s dementia	*p*
	HR (95% CI)		HR (95% CI)		HR (95% CI)	
Physical activity						
Unadjusted model	542 events		112 events		181 events	
Non-skiers (reference)	1		1		1	
Skiers	0.68 (0.58–0.81)	< 0.001	0.54 (0.37–0.80)	0.002	0.88 (0.66–1.18)	0.40
Model 1	533 events		112 events		177 events	
Non-skiers (reference)	1		1		1	
Skiers	0.63 (0.52–0.75)	< 0.001	0.49 (0.33–0.73)	< 0.001	0.74 (0.55–1.00)	0.052
Excluding dementia cases < 5 years						
Unadjusted model	483 events		104 events		169 events	
Non-skiers (reference)	1		1		1	
Skiers	0.78 (0.65–0.93)	0.005	0.61 (0.41–0.91)	0.014	0.94 (0.69–1.27)	0.68
Model 1	477 events		104 events		166 events	
Non-skiers (reference)	1		1		1	
Skiers	0.68 (0.57–0.82)	< 0.001	0.54 (0.36–0.80)	0.002	0.78 (0.57–1.07)	0.12

Association between physical activity and incident dementia in the Vasaloppet cohort, based on participation in a long-distance ski race (skiers) compared to non-skiers. Cox regression models showing hazard ratio (HR) with 95% confidence interval (CI) for risk of all-cause dementia, vascular dementia, or Alzheimer’s dementia, respectively. Model 1 adjusted for age, sex, and education

### Higher physical activity was associated with reduced risk of vascular dementia but not Alzheimer’s dementia in the MDCS

Demographics for all participants can be seen in Table 3. Participants were followed for a median of 20 years (IQR 19–22) from baseline and 15 years (IQR 14–17 years) from the reinvestigation. Based on the diagnostic review process, 1375 individuals were diagnosed with dementia during the follow-up period. Out of these, 300 (22%) were classified as VaD and 834 (61%) were classified as AD, out of which 436 were classified as pure AD and 398 as AD with concomitant cerebrovascular disease. In age-, sex-, and education-adjusted Cox regression models (model 1), higher physical activity score, modeled linearly, reduced the risk of developing VaD (HR 0.81 per SD increase, 95% CI 0.72–0.93), but not all-cause dementia (HR 0.96 per SD increase, 95% CI 0.91–1.02) nor AD (HR 1.03 per SD increase, 95% CI 0.97–1.09). In the fully adjusted model (model 2), the results were robust for VaD (HR 0.83 per SD increase, 95% CI 0.73–0.95). There was still no significant association between physical activity score and incident all-cause dementia (HR 0.97 per SD increase, 95% CI 0.92–1.02) or AD (HR 1.03 per SD increase, 95% CI 0.97–1.10) after full adjustments (model 2). When the population was categorized based on tertiles, high physical activity decreased the risk of developing VaD, even when we adjusted for multiple confounders (Table 4). We found no significant associations between physical activity categories and incident all-cause dementia or AD (Table 4). These results were not altered when cases who developed dementia within the first 5 years of the reinvestigation were excluded (Table 4). Further, we found no significant association between physical activity and pure AD (HR 1.06 per SD increase, 95% CI 0.98–1.14) nor between physical activity and AD with concomitant cerebrovascular disease (HR 1.04 per SD increase, 95% CI 0.92–1.17) in fully adjusted models (model 2).

**Table 3:** Characteristics of the MDCS population at baseline investigation (1991–1996)

	All	Low physical activity group	Intermediate physical activity group	High physical activity group
	*n* = 20,639	*n* = 6882	*n* = 6882	*n* = 6875
Characteristics at baseline	Median (IQR) or *n* (%)	Median (IQR) or *n* (%)	Median (IQR) or *n* (%)	Median (IQR) or *n* (%)
Age at baseline, years	57.5 (51.0–63.8)	57.0 (50.9–63.7)	57.1 (50.8–63.4)	58.3 (51.6–64.2)***
Women	12,460 (60)	4205 (61)	4335 (63)*	3920 (57)***
Education				
Primary/elementary school (≤ 8 years)	8159 (40)	3041 (44)	2515 (37)***	2603 (38)***
Secondary school/high school (9–12 years)	7449 (36)	2387 (35)	2568 (37)	2494 (36)
Higher education/university (≥ 13 years)	5001 (24)	1443 (21)	1793 (26)	1765 (26)
Smoking, ever	12,573 (61)	40,239 (62)	4151 (60)	4183 (61)
Systolic blood pressure, mmHg	140 (126–152)	140 (128–152)	140 (126–150)**	140 (126–152)
Diastolic blood pressure, mmHg	85 (80–90)	85 (80–90)	85 (80–90)**	85 (80–90)**
Body mass index, kg/m^2^	25.2 (22.9–27.7)	25.6 (23.2–28.3)	25.0 (22.8–27.5)***	25.0 (22.9–27.4)***
Alcohol, g/day	7.6 (1.9–15.6)	6.8 (1.3–15.3)	7.8 (2.3–15.7)***	8.1 (2.3–15.9)***
Physically heavy work	7659 (38)	2613 (39)	2444 (36)**	2602 (38)
Physical activity score combined	13,300 (8460–19,785)	6720 (4589–8460)	13,304 (11602–15,076)***	23,320 (19790–29,050)***
Cardiovascular disease	543 (2.6)	205 (3.0)	166 (2.4)*	172 (2.5)
Diabetes mellitus	790 (3.8)	305 (4.4)	235 (3.4)**	250 (3.6)*
Blood pressure-lowering medication	3568 (17)	1323 (19)	1177 (17)**	1068 (16)***
Lipid-lowering medication	629 (3.0)	207 (3.0)	205 (3.0)	217 (3.2)
*APOE*-ε4 carriers^a^	3306 (30)	1146 (31)	1055 (30)	1105 (30)
Dementia diagnoses at follow-up	*N* events (incidence rate/1000 person-years)			
All-cause dementia	1375 (4.7)	455 (4.8)	460 (4.7)	460 (4.7)
Vascular dementia	300 (1.0)	112 (1.2)	101 (1.0)	87 (0.9)
Alzheimer’s dementia	834 (2.9)	266 (2.8)	271 (2.8)	297 (3.0)
Age at dementia diagnosis	80.0 (75.7–83.7)	79.7 (75.8–83.2)	80.2 (75.7–84.1)	80.3 (75.8–84.1)

Characteristics of the MDCS population at baseline investigation (1991–1996) for the total cohort, and by physical activity tertiles. Blood pressure and body mass index were measured at the baseline investigation in the Malmo Diet and Cancer Study. Cardiovascular disease (coronary disease or stroke) and diabetes mellitus (type 1 or 2) were derived from hospital registries at baseline. Dementia diagnoses were derived from registries and validated in e-charts. All other data was self-reported, derived from the baseline questionnaire. Group differences between participants in the lowest physical activity group compared to intermediate and high respectively were estimated with Mann-Whitney *U* test (numeric variables) and Pearson’s *χ*^2^ test (categorical variables). Only significant differences are noted in the table

***p < 0.001, **p < 0.01, *p < 0.05

^a^Data on 10,971 participants (53% of the study cohort)

**Table 4:** Association between midlife physical activity and incident dementia in the MDCS cohort

	All-cause dementia	*p*	Vascular dementia	*p*	Alzheimer’s dementia	*p*
	HR (95% CI)		HR (95% CI)		HR (95% CI)	
Physical activity						
Model 1	1373 events		300 events		832 events	
Low (reference)	1		1		1	
Intermediate	0.99 (0.87–1.12)	0.84	0.87 (0.66–1.14)	0.30	1.01 (0.85–1.19)	0.95
High	0.90 (0.79–1.02)	0.11	0.63 (0.48–0.84)	0.002	1.04 (0.88–1.23)	0.64
Model 2	1341 events		293 events		815 events	
Low (reference)	1		1		1	
Intermediate	0.97 (0.85–1.11)	0.68	0.88 (0.67–1.16)	0.36	0.98 (0.82–1.16)	0.79
High	0.90 (0.79–1.03)	0.11	0.65 (0.49–0.87)	0.003	1.03 (0.87–1.22)	0.75
Excluding dementia cases < 5 years						
Model 1	1204 events		270 events		714 events	
Low (reference)	1		1		1	
Intermediate	1.02 (0.89–1.18)	0.75	0.92 (0.69–1.22)	0.55	1.06 (0.88–1.28)	0.54
High	0.95 (0.83–1.09)	0.47	0.65 (0.48–0.88)	0.005	1.14 (0.95–1.37)	0.16
Model 2	1172 events		263 events		697 events	
Low (reference)	1		1		1	
Intermediate	1.01 (0.88–1.17)	0.85	0.93 (0.70–1.24)	0.63	1.04 (0.86–1.25)	0.71
High	0.96 (0.83–1.10)	0.53	0.66 (0.49–0.90)	0.008	1.14 (0.95–1.37)	0.16

Association between midlife physical activity and incident dementia in the MDCS cohort, based on self-reported physical activity at two different occasions in midlife categorized as low, intermediate, or high activity group. Cox regression models showing hazard ratio (HR) with 95% confidence interval (CI) per physical activity group for risk of all-cause dementia, vascular dementia, or Alzheimer’s dementia, respectively. Number of events per model is presented for transparency, since we used complete case analyses. Model 1 adjusted for age, sex, and education. Model 2 adjusted for age, sex, education, smoking, systolic blood pressure, body mass index, alcohol consumption, diabetes, cardiovascular disease, blood pressure-lowering medication, lipid-lowering medication, and physically heavy work

Data on *APOE* genotype was available in a subpart of the MDCS cohort (*n* = 10,971), and 3306 participants (30%) were hetero- or homozygote *APOE-ε4* carriers. Three hundred five *APOE-*ε4 carriers (9.1%) were diagnosed with AD during the study period, compared to 2.6% among non-carriers and 4.6% in the total cohort (with available *APOE* data). When *APOE-*ε4 status was entered as a dichotomous covariate in the Cox regression models, the results per SD increase in physical activity score were not affected for any of the outcome variables (all-cause dementia, VaD, or AD) (data not shown). There was no significant interaction between *APOE*-ε4 and physical activity for any of the dependent variables (*p* = 0.68 for AD, *p* = 0.40 for vascular dementia, and *p* = 0.32 for all-cause dementia). When the population was stratified based on *APOE-*ε4 carrier status, physical activity did not affect the risk of developing AD among *APOE-*ε4 carriers (HR 1.00 per SD increase, 95% CI 0.89–1.13), nor among non-carriers (HR 1.04 per SD increase, 95% CI 0.92–1.16) in fully adjusted models (model 2).

By the end of the follow-up, 5220 individuals (25%) in the MDCS cohort were deceased, and among individuals without a dementia diagnosis, this number was 23%. In analyses treating death as a competing risk event, the association between physical activity and VaD was attenuated (fully adjusted HR 0.88 per SD increase, 95% CI 0.74–1.04 and HR 0.74 for the highest vs lowest physical activity group, 95% CI 0.56–0.98). There was still no association between physical activity and all-cause dementia (fully adjusted HR 1.00 per SD increase, 95% CI 0.95–1.05), but higher physical activity indicated a borderline increased risk of AD (fully adjusted HR 1.05 per SD increase, 95% CI 1.00–1.10), though not significant when modeled categorically (fully adjusted HR 1.13 for the highest vs lowest physical activity group, 95% CI 0.95–1.33).

Finally, to address attrition bias, we also performed analyses including all individuals who provided data on physical activity at baseline (*n* = 28,360), thus only assessing physical activity *once* in midlife. Still, no association was found between physical activity and incident all-cause dementia nor AD in either model 1 or 2 (all *p* > 0.20 per SD increase in physical activity score, data not shown). There was a significant association between physical activity at baseline and incident VaD in model 1 (HR per SD increase 0.87, 95% CI 0.78–0.96) and in model 2 (HR per SD increase 0.89, 95% CI 0.80–0.99).

### Physical activity does not protect against Alzheimer pathology in Alzheimer’s disease mice

**Fig. 3:**
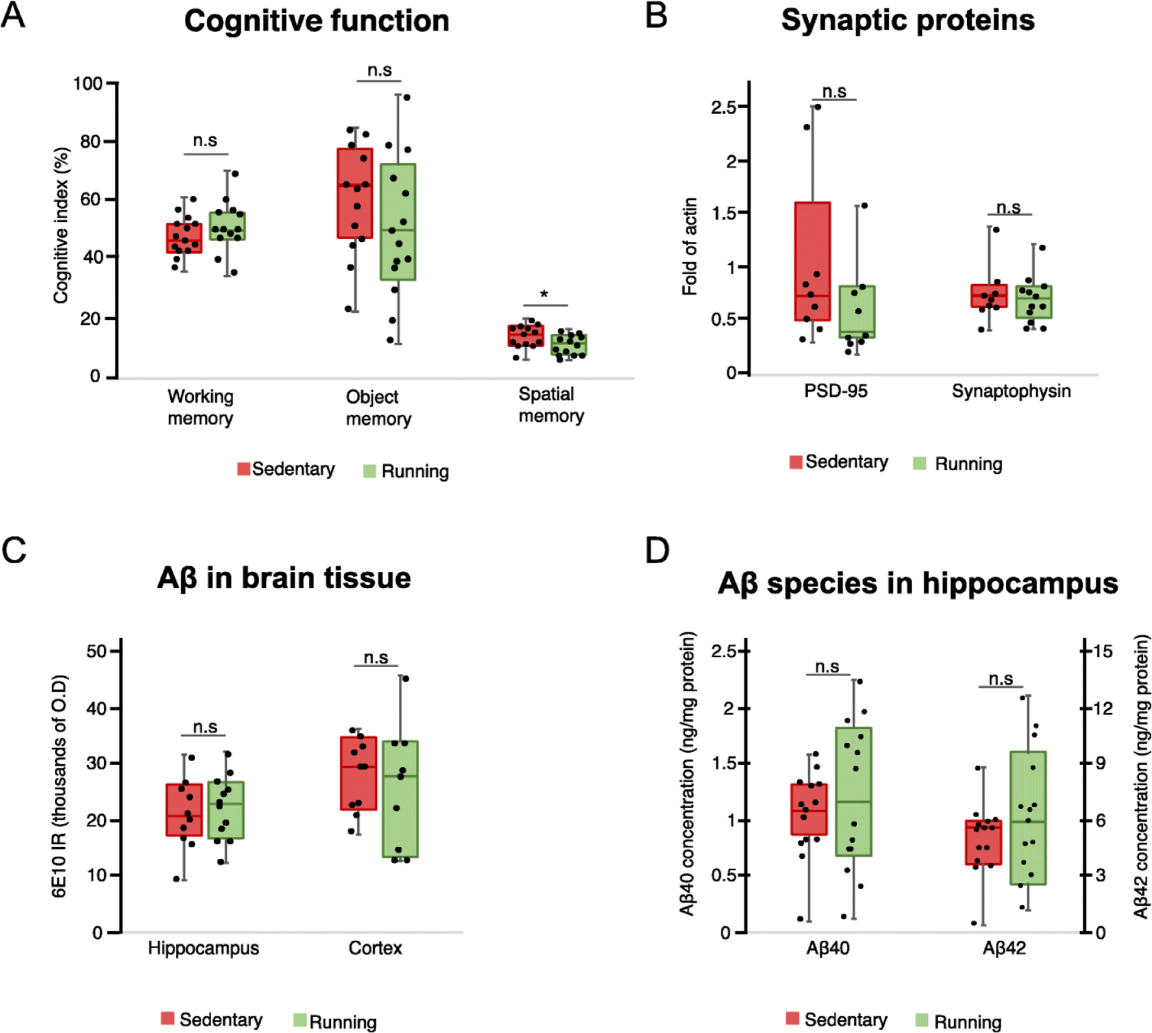
The effect of running on AD pathology in the 5xFAD mouse model. The effect on cognitive function (**a**), synaptic proteins (**b**) in the hippocampus, amyloid-β levels in the cortex and hippocampus (**c**), and Aβ-species in the hippocampus (**d**). Box plot represents the median values for each group with interquartile ranges and error bars indicating the minimum and maximum. *p < 0.05 in Mann-Whitney *U* test. For cognitive tests, *n* = 13–14 in each group; for amyloid-β and synaptic proteins, *n* = 9–14 in each group

Running did not affect the object memory (*p* = 0.21) or working memory (*p* = 0.38) (Fig. 3a). However, running mice had reduced spatial memory as they entered the new arm of the maze less frequently compared to sedentary mice (*p* = 0.03) (Fig. 3a). The levels of the synaptic proteins PSD-95 (*p* = 0.09) and synaptophysin (*p* = 0.79) in the hippocampus were not affected by running (Fig. 3b). Furthermore, the levels of amyloid-β did not differ between the running and sedentary mice, neither as measured by immunohistochemistry in the hippocampus (*p* = 0.77) or cortex (*p* = 0.40) (Fig. 3c and Additional file 1: Figure S1), nor as measured by ELISA in the hippocampus (*p* = 0.46 and *p* = 0.44 for Aβ40 and Aβ42 respectively, Fig. 3d).

## Discussion

Our study setup offered a unique possibility to study the effect of midlife physical activity on the development of different forms of dementia in very large study populations over long time periods. We found physical activity to be associated with lower incidence of VaD, but not AD, in both our epidemiological study populations. In addition, individuals carrying *APOE-*ε4, did not exhibit any specific beneficial protection from physical activity on the development of AD. The lack of protective effect of physical activity on the development of AD was also seen in an experimental setup subjecting AD transgenic mouse to voluntary wheel running.

The effect of physical activity on all-cause dementia differed in our study cohorts, in line with inconsistent results from previous studies [15, 17, 19, 20]. This might be due to the fact that all-cause dementia constitutes different underlying pathologies, identifying the need to differentiate between dementia subtypes. Indeed, for VaD and AD, our results were consistent in both cohorts. In line with a meta-analysis [12], we found physical activity to be associated with a lower incidence of VaD, presumably resulting from improved cerebral perfusion and reduction of cerebrovascular pathology [45]. In an attempt to use a more objective measure of physical activity, we stratified skiers based on the speed of race accomplishment. Interestingly, physically well-trained skiers had a lower incidence of VaD compared to less well-trained skiers, which further strengthen our results. Many previous studies suggest a beneficial effect of physical activity on the incidence of AD specifically [10, 19, 20, 46], but this was not confirmed in our study together with others [15, 18, 47]. Reasons for discrepancies between these studies may be that physical activity reduces cerebrovascular comorbidity in individuals with AD and thereby delays the onset of cognitive symptoms, rather than affecting AD pathology per se. Joint pathologies (generally concurrent cerebrovascular disease) are common in individuals diagnosed with AD [48]. Hence, studies that do report significant associations between physical activity and AD may represent effects that lower the cerebrovascular burden and thus postpone the onset of cognitive symptoms due to AD rather than affecting the specifc AD pathology per se. Studies using AD biomarkers and MRI as an outcome, rather than clinical dementia diagnoses, may help elucidate the specific effects. In recently published clinical studies, physical activity did not affect amyloid-β levels in the cerebrospinal fluid [49], but still resulted in improved cognition [7]. Further, physical inactivity was not associated with amyloid-β deposition measured with PET [50]. Another possible explanation to the beneficial effects of physical activity on dementia incidence shown in previous studies is the study setup. Lack of exclusion of participants developing dementia soon after physical activity assessment may increase the risk that some of them are affected by reverse causation, where reduced physical activity may be caused by cognitive decline and preclinical dementia symptoms [15, 17]. Indeed, when studies with follow-up time ≥ 10 years were assessed separately in a meta-analysis, the impact of physical activity on dementia was more conservative [11]. Consistently, physical activity was associated with reduced risk for dementia with cerebrovascular disease, but not AD, in a recent study following 800 women over 44 years [51]. In the present study, we tried to limit reverse causation by excluding individuals who developed dementia within 5 years of the ski race or the physical activity assessment. Further, publication bias may have influenced the prevailing literature, since a large number of smaller studies showed larger-than-average effects [11].

Lately, intervention trials have been carried out to test if physical activity may reduce cognitive decline and dementia. The overall effects seem limited [3, 4], but one study with a multi-domain intervention found beneficial effects on cognitive performance [5]. In the study with the longest follow-up (mean 6.7 years), the risk of developing non-AD dementia was significantly reduced, with a trend towards protection against VaD specifically [3]. Moreover, when assessing the intervention effects of physical activity on cognitive performance in *APOE*-ε4 carriers and non-carriers separately, there was no effect difference depending on the genetic risk [52], which agrees with the present study.

In experimental settings, we have not been able to find any data on the effect of physical activity on pathological processes in animal models of VaD. However, the effects of exercise on AD pathology have been thoroughly studied in mice [25]. Many studies report the ability of exercise to improve cognition in aged wild-type mice as well as transgenic AD mice [25]. Nevertheless, some studies show no effect of exercise on cognition in transgenic AD models [27, 33] and some experimental studies can be biased by chronic stress, as reported by us [41]. In the present study, voluntary physical activity did not improve cognition in transgenic 5xFAD mice. Furthermore, physical activity did not reduce the levels of amyloid-β or formation of plaques, which is congruent with some previous studies [25, 27]. Important parameters to consider for the discrepancies between studies are the duration and timing of the exercise interventions and sample collection. As noted by Ryan et al., longer durations of exercise interventions are needed to investigate the long-term effects of an active lifestyle [25]. Many published studies have limitations in the timings and durations in order to study the effect of a long-term active lifestyle from middle age and onwards [25]. We initiate the exercise at an age of 2 months, just before the onset of Aβ pathology. Further, our intervention lasts for as long as 6 months, until the mice are 8 months old, an age with fully developed pathology. Given the genetically driven pathology in most transgenic AD models, the effects of exercise investigated might not be fully transferable to late-onset AD.

Limitations of the study include that physical activity was self-reported in the MDCS cohort, which introduces subjectivity into the estimation. We tried to compensate this with the use of a validated physical activity score [33] and by using data from two separate time points (5 years apart), thereby estimating the degree of physical activity over an extended time period in midlife. Further, we assume there is a healthy selection bias considering that individuals included in MDCS were generally healthier and more physically active at baseline than those excluded due to lack of data (see “Attrition” in the MDCS methods section). This may underestimate any true associations, but this was partly accounted for in sensitivity analyses where we included all individuals with baseline data on physical activity (only one assessment), thus minimizing attrition during follow-up. Still, no significant association was found for all-cause dementia nor AD. The association between physical activity and vascular dementia was weaker in the analyses with *one* physical activity assessment (see results for MDCS). This may be due to the possibility that the potential effects of physical activity require an active lifestyle during a prolonged period, better reflected when physical activity was reported twice. In the Vasaloppet cohort, we lack data on physical activity among non-skiers and thereby include physically active individuals in the reference category as well, which may attenuate the true association. Skiers were considered physically active based on the assumption that it is necessary to undergo regular physical training in order to complete such a demanding long-distance race, and previous studies have indeed showed that this is the case [30]. This may induce bias dependent on other confounders, such as diet, BMI, and smoking habits. Since this information cannot be found in the Swedish registries, we could not adjust for these potential confounders. Still, the results of the association between physical activity and incidence of VaD and AD were in accordance with those from the fully adjusted model in the MDCS cohort. Nevertheless, we were able to adjust for age, sex, and education in the statistical models in the Vasaloppet. In addition, we clearly demonstrated that faster skiers had reduced incidence of VaD but not AD, implicating that the associations seen can be attributable to physical fitness level per se. In the MDCS, the study protocol provided data on several possible confounders that were included in the analyses. Lastly, the use of register-based diagnoses can be considered a limitation. All dementia diagnoses were derived from hospital registries, which most likely underestimates the true incidence. However, the Swedish National Patient Register covers 99% of all hospital-based diagnoses, and both primary and secondary diagnoses are represented. Another explanation to the relatively low incidence of dementia within the Vasaloppet cohort is that the study design excluded individuals that were already diagnosed with a severe disease that could prevent them from being active at baseline. This was necessary in order to reduce the potential bias due to inability to participate in the ski race. Hence, this design is likely to result in a lower incidence number due to elimination of comorbidity. In Sweden as a whole, the incident rate of dementia is 2 cases per 1000 person-years (2017, Statistics Sweden). The incident rate in MDCS is around this number, mainly due to participants being older (around 58 years). In the Vasaloppet cohort, the incident rates are below this, mainly due to the exclusion of comorbidities and a low age at baseline (around 36 years). Finally, since we aimed to study differences between dementia subtypes, possible diagnostic misclassification needs to be acknowledged. Clinically derived diagnoses may be insufficiently characterized, and concordance between clinical and neuropathological diagnoses does vary [53]. Nevertheless, in the MDCS, over 80% of individuals with dementia attended specialized Memory Clinics, and all medical records and brain imaging were retrospectively reviewed to determine the type of dementia diagnosis.

Taken together, we used two very different study designs, one in which physical activity was measured in a more objective way (participation in long-distance ski race), and the other where it was subjectively measured (by a self-reported questionnaire). Still, both these study setups revealed concurrent results where physical activity was associated with a lower incidence of VaD but not AD, despite differences in strengths and limitations within the separate cohorts. This consistency likely reduces the risk that the found associations are driven by confounding factors.

## Conclusion

In conclusion, higher physical activity in midlife was associated with a lower incidence of VaD. No association between physical activity and AD was found, neither among individuals predisposed to develop AD by carrying the *APOE*-ε4 risk allele. Altogether, physical activity could be an important strategy to prevent the development of VaD, especially considering the lack of available treatments for this disease.

## Supplementary information

**Additional file 1: Figure S1.** Representative pictures of the 6E10 staining of cortex and hippocampus in sedentary and running mice respectively. Scale bar represents 100 μm. No differences were found between groups with the Mann-Whitney U-test.

## Data Availability

The data sets supporting the conclusions of this article can be made available upon request. MDCS data can be requested through an application to the MDCS steering committee. Vasaloppet database can be requested from Uppsala Clinical Research Center. Data used in the mouse model analyses can be requested through the corresponding author.

## Abbreviations

5xFAD: 5x familial Alzheimer’s disease
AD: Alzheimer’s disease
HR: Hazard ratio
IQR: Interquartile range
MDCS: Malmo Diet and Cancer Study
NPR: National Patient Register
VaD: Vascular dementia
