# Supplementary figures and images for: Midlife physical activity is associated with lower incidence of vascular dementia but not Alzheimer’s disease
Source: Alzheimers Res Ther. 2019 Oct 20;11:87. doi: 10.1186/s13195-019-0538-4 (PMC6802179; doi:10.1186/s13195-019-0538-4)

Figure S1.

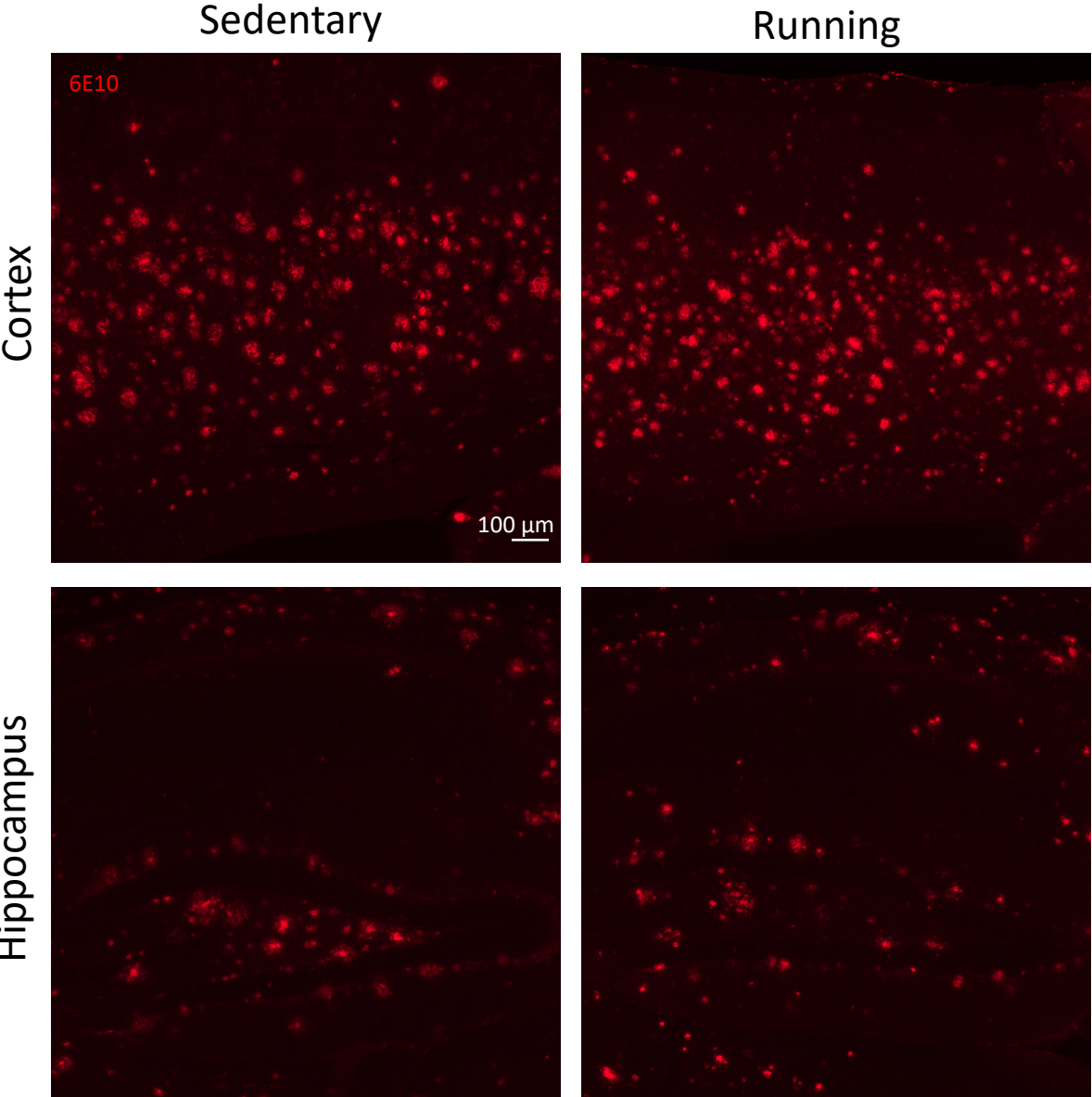

Supplement: Supplementary file 1 — Additional file 1: Figure S1. Representative pictures of the 6E10 staining of cortex and hippocampus in sedentary and running mice respectively. Scale bar represents 100 μm. No differences were found between groups with the Mann-Whitney U-test. [file 13195_2019_538_MOESM1_ESM.pdf]
